# Seasonal Volatile Emission Patterns of the Endemic New Zealand Shrub *Dracophyllum subulatum* on the North Island Central Plateau

**DOI:** 10.3389/fpls.2021.734531

**Published:** 2021-10-15

**Authors:** Evans Effah, D. Paul Barrett, Paul G. Peterson, Murray A. Potter, Jarmo K. Holopainen, Andrea Clavijo McCormick

**Affiliations:** ^1^School of Agriculture and Environment, Massey University, Palmerston North, New Zealand; ^2^Manaaki Whenua - Landcare Research, Massey University, Palmerston North, New Zealand; ^3^Department of Environmental and Biological Sciences, University of Eastern Finland, Kuopio, Finland

**Keywords:** Central Plateau, *Dracophyllum subulatum*, environmental variables, volatile organic compounds, warming, high temperature, native species

## Abstract

Volatile organic compounds (VOCs) produced by plants are essential indicators of their physiological response to environmental conditions. But evidence of natural variation in VOC emissions and their contributing factors is still limited, especially for non-cultivated species. Here we explored the natural volatile emissions of *Dracophyllum subulatum* Hook.f., an endemic shrub to the North Island Central Plateau of New Zealand, and determined some environmental factors driving the plant’s emissions. Volatile emissions of *D. subulatum* were measured on four separate occasions from December 2017 to September 2018 using the “push-pull” headspace sampling technique and analyzed using gas chromatography-mass spectrometry (GC-MS). *D. subulatum* was classified based on the volatiles measured on each sampling occasion using linear discriminant analysis (LDA). On each sampling occasion, we also recorded and compared ambient air temperature, herbivory damage, total soil nitrogen (N), available phosphorus (P), potassium (K), and soil moisture content. The relationship between environmental variables that differed significantly between sampling occasions and volatile emissions were estimated using generalized linear models (GLMs). Based on VOCs measured on each sampling occasion, we were able to distinguish different chemical profiles. Overall, we found that total emission and the relative proportions of all major chemical classes released by *D. subulatum* were significantly higher during summer. The GLMs reveal that differences in environmental factors between the four sampling occasions are highly associated with changing emissions. Higher temperatures in summer had a consistently strong positive relationship with emissions, while the impacts of soil moisture content, P and K were variable and depended on the chemical class. These results are discussed, particularly how high temperature (warming) may shape volatile emissions and plants’ ecology.

## Introduction

Volatile organic compounds (VOCs) produced by plants are typically lipophilic molecules with high vapor pressure. Hence, they can easily cross membranes and be released into the surrounding environment when diffusion barriers are lacking (Pichersky et al., 2006). As a result, copious quantities of constitutive and stress-induced plant volatiles are released into the atmosphere. Consequent to their release, VOCs act as critical mediators for within plant, between plants, plant-insect and plant-microbe communication (Holopainen, 2004; Dudareva et al., 2006; Heil, 2010; Ninkovic et al., 2020), which play a key role in shaping plant communities (Kegge and Pierik, 2010; Effah et al., 2019).

The composition of volatile bouquets is species-specific (Bracho-Nunez et al., 2011; Malik et al., 2018) yet influenced by biotic and abiotic environmental variables (Holopainen and Gershenzon, 2010; Clavijo McCormick, 2016; Copolovici and Niinemets, 2016). Herbivory has frequently been linked to increased emissions of terpenoids (Clavijo McCormick et al., 2019; Effah et al., 2020d) and lipoxygenase (LOX) products that account for over 50% of damage-induced volatiles (Holopainen, 2004). Damage-induced volatiles reduce herbivore loads and increase resistance to future attacks in natural systems (Kessler and Baldwin, 2001; Kessler et al., 2006; Hu et al., 2019). However, VOC emissions in response to herbivory can vary depending on the attacker’s density and identity, season, and the plant’s phenology (Clavijo McCormick et al., 2012, 2014; Effah et al., 2020d).

Belowground components like soil moisture and nutrients also have a strong influence on plant volatile emissions. However, the response of plants to water availability differs considerably between species. For instance, some plants release more VOCs in response to drought (Copolovici et al., 2014) while others significantly reduce their emissions (Lavoir et al., 2009), and outcomes are even more ambiguous for prolonged or different drought intensities (Wu et al., 2015; Pagadala Damodaram et al., 2021). Similarly, the impact of soil nutrients on VOC emissions depends on the nature of chemical compound but also plant species-specific and may even vary for different types of nutrients (Chen et al., 2008; Holopainen and Gershenzon, 2010; Effah et al., 2020a,c).

In contrast, studies investigating relationships between temperature and plant volatile emissions have shown more consistent results, with most demonstrating a strong positive relationship between high temperature (warming) and VOC emissions (Kramshøj et al., 2016; Lindwall et al., 2016; Tiiva et al., 2017; Effah et al., 2020c; Rinnan et al., 2020). This relationship can be explained by the direct effect of temperature on enzyme activities, biosynthetic pathways, stomatal conductance, and physicochemical properties of compounds; and indirectly through antagonistic effects on other variables like drought and plant phenology (Peñuelas and Staudt, 2010; Possell and Loreto, 2013).

In nature, environmental factors, including temperature, soil nutrients, and herbivore loads, fluctuate between seasons (Shiojiri and Karban, 2008; Omer et al., 2018; Effah et al., 2020c), which often correspond to changes in plants’ physiological status. Therefore, measuring VOCs can be a good indicator of plant responses to environmental changes associated with different seasons and provide valuable information about how plant communication changes over time. This is increasingly important since most of these environmental parameters are changing globally due to climate change (Collins et al., 2013; Pachauri et al., 2014). However, information about how these climate components influence plant volatile emissions, particularly for endemic plant species, is still limited. Such information is critical to predicting alterations in plant volatiles’ ecological roles, their interference with other atmospheric processes such as cloud formation and reacting with O_3_, and will increase our understanding of the impact of climate change on plant communication networks.

**FIGURE 1:**
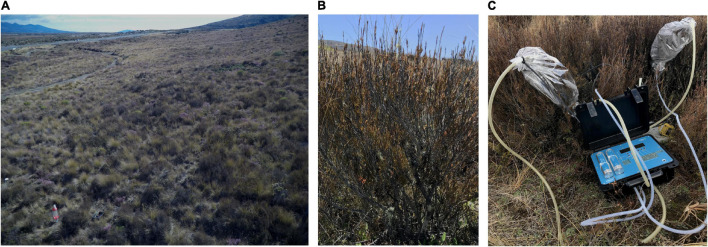
Images of part of the sampling location **(A)**, *Dracophyllum subulatum* plant **(B)**, and volatile collection procedure **(C)**.

Here, we explored seasonal VOC emission by the New Zealand native plant *Dracophyllum subulatum* Hook.f., (Ericaceae). This woody shrub is endemic to the North Island of New Zealand and an archetypal species of the frost flats found on the North Island Central Plateau that thrives in the cool climate and infertile volcanic soils (Smale, 1990; Smale et al., 2011). This evergreen plant typically grows up to 0.3–2.0 m tall and has paniculate inflorescence. The adult narrow grass-like leaves are 10–48 mm long (Figure 1) and have stomata only present on the abaxial surface. *D. subulatum* has 2–6 inflorescences, and its tiny reticulate seeds are dispersed by wind (Venter, 2009; de Lange, 2021).

Unfortunately, the occurrence of *D. subulatum* in this region has been reduced considerably by conversion to pasture, the planting of exotic invasive conifers and invasion by aggressive alien weeds such as *Calluna vulgaris* and *Cytisus scoparius* (Chapman and Bannister, 1990; Buddenhagen, 2000; Smale and Fitzgerald, 2004), which also impact the arthropod diversity associated with this plant (Effah et al., 2020b). Nevertheless, *D. subulatum* spp. are an important component of the unique New Zealand Flora, having associations with native fauna (Eagle, 2006). The plant retains its foliage throughout the year and has a broad flowering and fruiting period (de Lange, 2021), serving as a food source for various animals.

Previous studies on the ecology of *D. subulatum* demonstrate that the plant is resilient to fire and even suggest that occasional fires in the past bolstered its dominance by initiating new populations (Smale, 1990; Smale and Fitzgerald, 2004; Smale et al., 2011). *Dracophyllum* species are highly flammable and popularly referred to as “turpentine shrub” (Smale et al., 2011; Cui et al., 2020). Turpentine is composed of terpenes, with monoterpenes α- and β-pinenes being the main components (Mercier et al., 2009). The plant, therefore, likely produces other several secondary compounds that, along with terpenes, contribute to its flammability.

This study aimed to investigate the VOCs emitted by *D. subulatum* and explore the factors driving their emissions in nature. We measured the emissions of *D. subulatum* at different sites on the North Islands’ Central Plateau. VOCs were measured on four different occasions from December 2017 to September 2018 over several seasons. On each sampling occasion, we also collected information about biotic (herbivore damage) and abiotic factors (ambient temperature, soil moisture, and soil nutrients; N, P, K) and determined their relationship with the plant’s volatile emissions.

## Materials and Methods

### Study Area and Experimental Procedure

The study was conducted on the Central Plateau of the North Island, New Zealand. This sub-alpine area has a cool climate, with low fertility volcanic ash soils, which support a relatively homogenous secondary successional shrubland plant community (Leathwick and Mitchell, 1992; Rogers, 1994). Some common plants in this region include natives like *Chionochloa rubra*, *Leptospermum scoparium*, *D. subulatum*, and introduced plants such as *C. vulgaris* and *C. scoparius* (Chapman and Bannister, 1990; Rogers, 1994; Buddenhagen, 2000). For this study, we selected four sites in the Waiouru Military Training Area, which lies within the Central Plateau. These sites, which lay along the Desert Road, were at high altitudes and had a natural population of *D. subulatum* occurring with other plants. For instance, the sites were *D. subulatum* dominated or a mix of predominant *D. subulatum* with either *L. scoparius, C. vulgaris* or *C. cytisus* (Supplementary Table 1). We visually inspected target *D. subulatum* to ensure that they were of similar size and growth stage. At each location, volatile emissions of selected *D. subulatum* plants were measured, and data for some environmental parameters known to influence plant volatile emissions (Holopainen and Gershenzon, 2010; Clavijo McCormick, 2016; Effah et al., 2020c) were also collected. We collected data on four separate occasions from December 2017 to September 2018, covering early summer (ES) (5 – 13 December 2017), late summer (LS) (15 – 26 February 2018), late autumn (LA) (1 May – 1 June 2018), and winter (W) (26 August – 11 September 2018).

### Measuring *Dracophyllum subulatum* Volatile Emissions

The aboveground volatiles emitted by *D. subulatum* was collected by enclosing a portion of foliage in new oven bags (AWZ products) during each sampling occasion (Figure 1). A portable volatile collection system (PVAS22; Volatile Assay Systems, Rensselaer, NY, United States) connected with PTFE tubes was used to pump carbon-filtered air into the bags (0.85 L min^–1^) and simultaneously pull air out (0.80 L min^–1^) through volatile collection traps containing 30 mg HayeSep Q adsorbent (Volatile Assay Systems, Rensselaer, NY, United States). Volatiles of each plant were collected for 2 h, and foliage enclosed in oven bags was excised after VOC sampling, oven-dried (60°C for 72 h) and used to quantify emissions per dry weight (DW) (g). VOCs were collected on dry and sunny days after 9:30 am and before sunset during each sampling occasion. We reduced the bias of sampling time by randomly collecting samples simultaneously from each site and pooled them for the final analysis.

After sampling, the volatile collection traps were wrapped with aluminum foil and stored in a portable cooler for transport. All samples were eluted within 24 h. Volatile compounds were eluted from collection traps with 200 μL of 95% hexane with 10 ng/mL nonyl acetate (Sigma Aldrich, Buchs, Switzerland) added as an internal standard. The collected samples were analyzed using gas chromatography-mass spectrometry (QP2010; GCMS Solution version 2.70, Shimadzu Corporations, Kyoto, Japan), with a 30 m × 250 μm × 0.25 μm TG-5MS capillary column (Thermo Fisher Scientific, Waltham, MA, United States). Helium was the carrier gas and supplied at 53.5 kPa pressure, total flow 14.0 mL/min, linear velocity 36.3 cm/s and purge flow 3.0 mL/min. The temperature programme was: initial oven temperature of 50°C held for 3 min, then increased to 95°C at a rate of 5°C/min and then 15°C/min to 240°C. Using the gas chromatography-mass spectrometry (GC-MS) postrun analysis software supplied by Shimadzu Corporation, compounds were identified by comparing target spectra to the mass spectra library from the National Institute of Standards and Technology (NIST) and confirmed using commercial standards, when available. Chromatographic analyses were performed in scan mode and peaks quantified relative to the internal standard, then divided by the DW of enclosed foliage and sampling time (h) to estimate emissions per DW per hour. We collected compounds in the air of oven bags without plant (blanks) at each season and excluded these compounds from the data. VOCs were measured from the same *D. subulatum* plants in each season.

### Measuring Visible Herbivore Damage on *Dracophyllum subulatum*

Herbivore damage on foliage enclosed in oven bags during volatile collection was examined using a handheld magnifying glass before oven-drying. Damage marks were counted and divided by the DW of foliage as described by Effah et al. (2020a).

### Measurement of Abiotic Environmental Variables

Soil properties were determined by collecting and homogenizing 20 soil cores (15 cm deep × 3 cm diameter) from each of the four sites (replicates) on each sampling occasion. Soil moisture content was estimated gravimetrically and expressed as a percentage. Total nitrogen (N), available phosphorus (P) and potassium (K) were measured by a commercial laboratory as described by Effah et al. (2020a).

During each sampling occasion, ambient air temperature at each site was recorded by installing temperature data loggers (Tinytag, Gemini) 50 cm above ground level. Loggers were installed 10 days before the commencement of volatile collection and retrieved on the last sampling day.

### Data Analysis

The relative proportions of major chemical classes, total volatile emissions and environmental variables were compared between sampling occasions using the Kruskal–Wallis test and, when significant, followed by the Mann Whitney test for pairwise comparisons.

We used linear discriminant analysis (LDA) to classify *D. subulatum* based on the plant’s emissions on each sampling occasion, and the quality of separation was estimated using Wilk’s lambda (Wilks’ Λ). LDA was performed with the *Mass package* (Ripley et al., 2013) based on log-transformed (log10x + 1) data of individual volatile compounds identified.

The relationship between measured environmental variables and major chemical classes produced by *D. subulatum* was determined using Bayesian generalized linear models (GLMs) with normal likelihood and assuming priors were Gaussian distributed. Chemical classes were response variables, while environmental variables that differed significantly between seasons were used as predictors. All predictor variables were z-scored standardized before modeling. Models were performed using the *rstanarm package* with the default weakly informative priors (Goodrich et al., 2018; Muth et al., 2018). Each model was run for 1,00,000 iterations, with 10 thinning and 3 chains.

All statistical analyses were performed with R version 3.6.2.

## Results

### Volatile Emissions on Four Sampling Occasions

Forty-six volatile compounds were abundant in the headspace of *D. subulatum*. Most of the identified compounds were sesquiterpenes (39.13%), monoterpenes (19.57%), and fatty acid derivatives (15.22%), while aldehydes, other esters, and other alcohols constituted the remaining proportion (Table 1).

**TABLE 1:** List of volatile compounds identified from the air surrounding enclosed *Dracophyllum subulatum* foliage. Compounds grouped by their major chemical classes.

Code	Compound	Code	Compound	Code	Compound
	**Fatty acid derivatives**		**Sesquiterpenes**		**Other esters**
1	Hexyl acetate	17	(*E*)-α-bergamotene	35	3-methyl-1-butanol acetate
2	Hexanol	18	(*E*)-β-caryophyllene^i^	36	Ethyl hexanoate
3	(*E*)-2-hexenal	19	Aromadendrene	37	Ethyl octanoate
4	(*E*)-2-hexenyl acetate	20	Copaene	38	Hexyl 2-methylbutyrate
5	(*Z*)-2-hexenyl acetate	21	Eremophilene	39	Phenethyl acetate
6	(*Z*)-3-hexenyl acetate^i^	22	Germacrene D		
7	(*Z*)-3-hexenol^i^	23	Humulene^i^		**Aldehydes**
		24	Isoledene	40	Decanal
	**Monoterpenoids**	25	Valencene	41	Dodecanal
8	(*Z*)-β-ocimene	26	Zingiberene	42	Heptanal
9	Lemonol	27	α-amorphene	43	Nonanal
10	Limonene^i^	28	α-bourbonene	44	Octanal
11	Linalool^i^	29	α-cubebene		
12	Linalyl acetate	30	(*E*, *E*)-α-farnesene		**Other alcohols**
13	Perillene	31	α-panasinsene	45	Non-anol
14	Sabinene	32	β-cubebene	46	Octanol
15	α-pinene^i^	33	γ-cadinene		
16	β-pinene^i^	34	δ-cadinene		

*^*i*^Compounds verified by authentic standards.*

**FIGURE 2:**
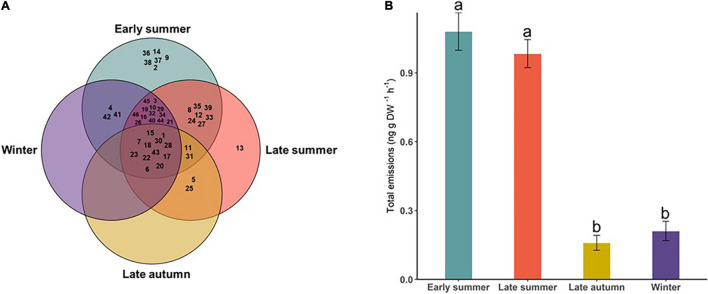
**(A)** Volatile compounds identified in the headspace of *D. subulatum* on sampling occasions. Numbers in the chart represent the codes assigned to compounds in Table 1. **(B)** Comparison of total volatile emissions of *D. subulatum* between the four sampling occasions (*n* = 25). Bars show log-transformed mean ± SE emissions. Different letters indicate significant differences between groups.

Some compounds were found only on a given sampling occasion, whereas others were measured from multiple occasions. For example, hexanol, lemonol, sabinene, ethyl hexanoate, ethyl octanoate, and hexyl 2-methylbutyrate were found only in ES, whereas perillene was only identified in LS. Other compounds were found on two or more sampling occasions (Figure 2A and Supplementary Figure 1). Overall, volatile emissions by *D. subulatum* were significantly higher during summer sampling compared with autumn and winter sampling (Kruskal–Wallis; *X*^2^ = 69.90, *df* = 3, and *P* < 0.001, Figure 2B).

**FIGURE 3:**
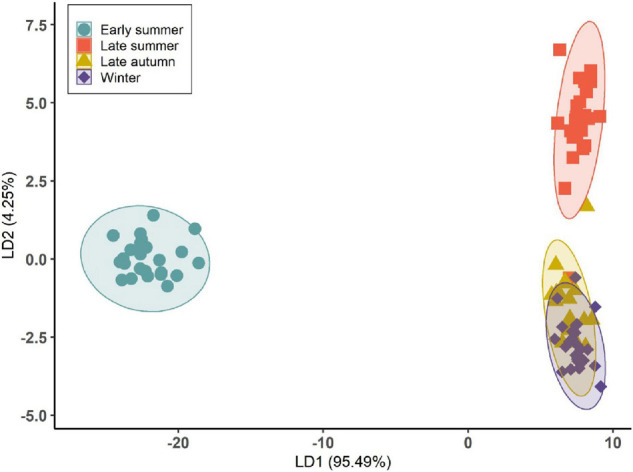
Linear discriminant analysis (LDA) based on the volatile compounds identified from *D. subulatum* on four different sampling occasions. Ellipse displaying 99% confidence interval (*n* = 25).

Using LDA, *D. subulatum* was classified based on the forty-six volatile compounds identified from the plant. The results showed a significant separation of groups across sampling occasions (Wilks’ Λ = 0.0005, *F*_3_,_96_ = 13.30, and *P* < 0.001). Emissions in summer were separated from autumn and winter in the first two linear discriminants, which accounted for 95.49 and 4.25% of the observed variance between the groups (Figure 3 and Supplementary Table 2).

**FIGURE 4:**
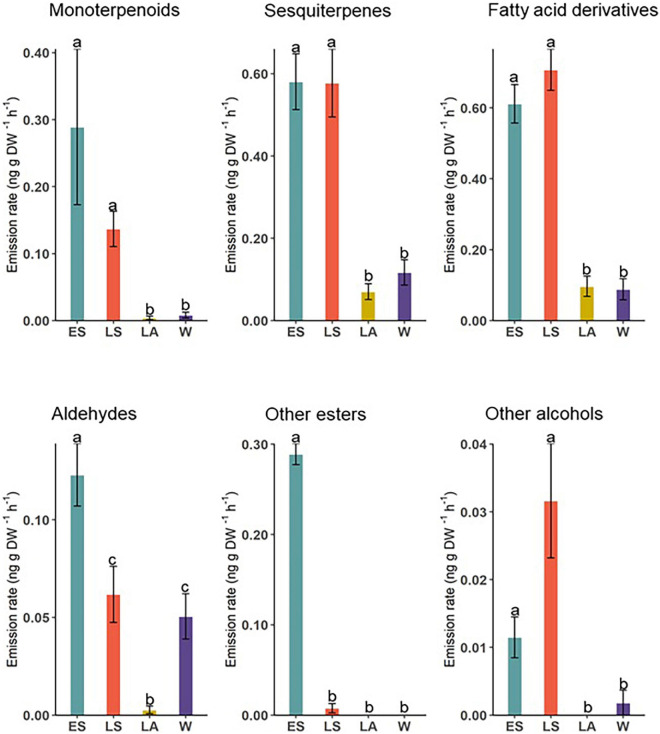
Comparison of major chemical classes identified from *D. subulatum* in early summer (ES), late summer (LS), late autumn (LA), and winter (W). Bars show log-transformed mean ± SE emissions. Different letters indicate significant differences between groups (*n* = 25).

Volatile compounds measured from *D. subulatum* were grouped into major chemical classes and their relative proportions compared between the four sampling occasions (Figure 4). The results showed significant differences in total monoterpenoids (Kruskal–Wallis; *X*^2^ = 49.60, *df* = 3, and *P* < 0.001), sesquiterpenes (Kruskal–Wallis; *X*^2^ = 50.13, *df* = 3, and *P* < 0.001), fatty acid derivatives (Kruskal–Wallis; *X*^2^ = 65.14, *df* = 3, and *P* < 0.001), aldehydes (Kruskal–Wallis; *X*^2^ = 45.68, *df* = 3, and *P* < 0.001), other esters (Kruskal–Wallis; *X*^2^ = 89.50, *df* = 3, and *P* < 0.001), and other alcohols (Kruskal–Wallis; *X*^2^ = 26.90, *df* = 3, and *P* < 0.001).

### Comparison of Measured Environmental Variables

**FIGURE 5:**
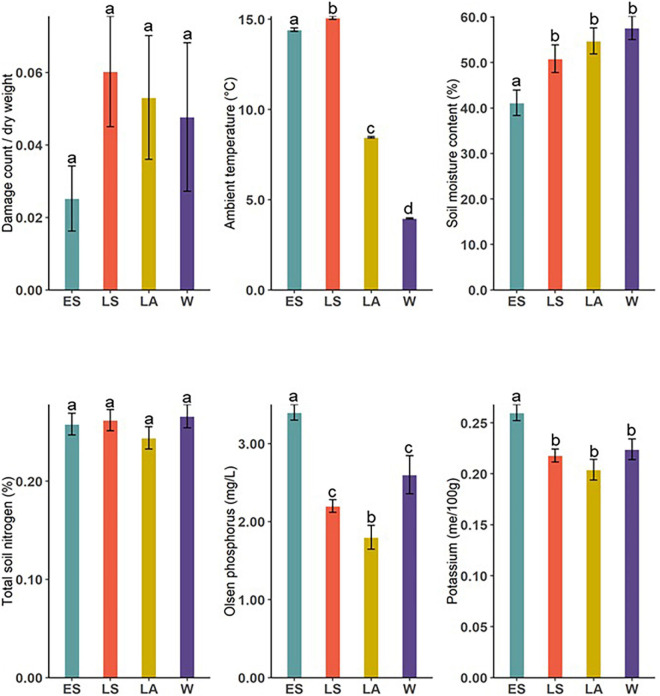
Comparison of measured environmental variables between ES, LS, LA, and W. Bars show mean ± SE of measured variables. Different letters indicate significant differences between groups.

We measured some biotic and abiotic variables at the sites and compared them between the four sampling occasions (Figure 5). There was a significant difference in ambient temperature (Kruskal–Wallis; *X*^2^ = 15,144, *df* = 3, and *P* < 0.001), soil moisture content (Kruskal–Wallis; *X*^2^ = 19.86, *df* = 3, and *P* < 0.001), available phosphorus (Kruskal–Wallis; *X*^2^ = 45.49, *df* = 3, and *P* < 0.001), and potassium (Kruskal–Wallis; *X*^2^ = 15.69, *df* = 3, and *P* = 0.001). Compared to other sampling occasions, herbivore damage on *D. subulatum* was slightly higher in LS but the difference was not significant (Kruskal–Wallis; *X*^2^ = 2.054, *df* = 3, and *P* = 0.561), whereas total soil nitrogen was not significantly different between sampling occasions (Kruskal–Wallis; *X*^2^ = 6.083, *df* = 3, and *P* = 0.108, Figure 5).

### Influence of Environmental Variables on Volatile Production

**FIGURE 6:**
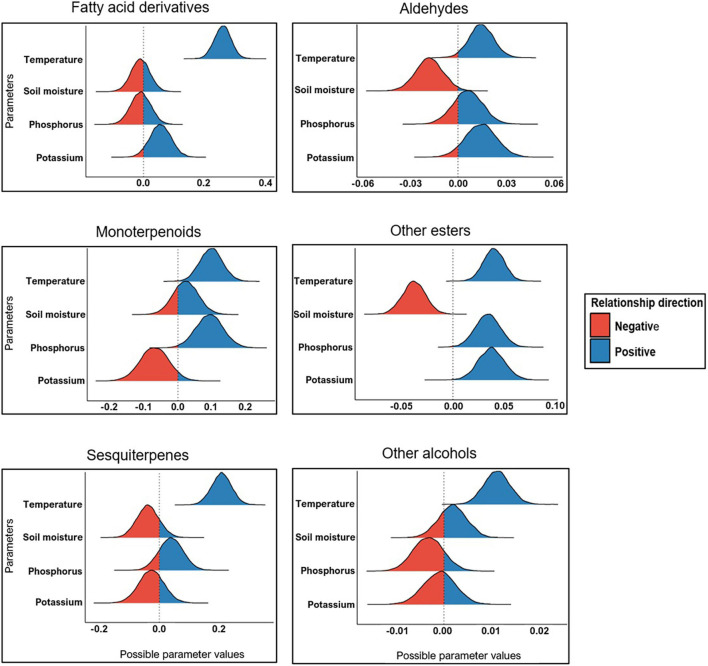
Relationship between environmental variables and major chemical classes. Bell shapes indicate the distribution of covariates (predictors) draws after the Markov chain Monte Carlo (MCMC) simulations. Distribution that is fully below or above the reference line on zero indicates a strong, consistent relationship, and colors (red or blue) show the direction of the relationship (either positive or negative).

Bayesian GLMs with various major chemical classes as response variables (Figure 3 and Table 1) and temperature, soil water content, phosphorus and potassium as predictors were used to elucidate the relationship between environmental factors and the production of *D. subulatum* volatiles. We selected these predictors since they varied significantly between the four sampling seasons (Figure 5). The results showed a consistently strong positive relationship between temperature and the production of all the major chemical classes. However, the relationship between soil moisture content, phosphorus and potassium was variable and depended on chemical classes (Figure 6 and Supplementary Table 3). Soil moisture content had a negative relationship with aldehydes, other esters and sesquiterpenes, while monoterpenoids had a weak positive association with this parameter. Phosphorus was positively associated with other esters, monoterpenoids and sesquiterpenes but was negatively related to other alcohols. Potassium also had a positive relationship with other esters, aldehydes, and fatty acid derivatives but was negatively associated with monoterpenoids and sesquiterpenes (Figure 6 and Supplementary Table 3).

## Discussion

Our findings show that *D. subulatum* Hook. f. is rich in terpenoids, including α- and β-pinenes, which may contribute to the popular generic name of “turpentine shrub” and its high flammability (Smale et al., 2011). Information about volatile storage and secretory structures such as glandular trichomes and resin ducts in *D. subulatum* is scarce. However, the enormous amount of terpenes observed in our study and the plant’s high flammability, as reported earlier, are typical of terpene-storing species (Llusià et al., 2006; Pausas et al., 2016; Della Rocca et al., 2017). Our results also reveal a clear seasonal pattern in VOC emissions, with total emissions ranging from 22.27 to 545.75 ng gDW^–1^ h^–1^ during winter and summer seasons, respectively. The findings further demonstrate that several environmental variables, including ambient temperature, soil water content, P and K availability, differ between sampling occasions, corresponding with the observed variable emissions. We acknowledge that plant phenology can influence volatile emissions, too, and suggest future studies to explore the phenology of the plant in relation to its volatile emission. Unfortunately, *D. subulatum*’s phenology is poorly understood, but it is known to retain its foliage through the winter and have broad flowering and fruiting periods (de Lange, 2021). Therefore, we will focus on the effect of environmental variables.

Ambient temperature was significantly higher during the summer months and corresponded with lower soil moisture content (Figure 5). This was also true when comparison was made separately for night- and daytime temperatures (Supplementary Figure 2). Temperature had a consistently strong positive relationship with the production of monoterpenoids, sesquiterpenes, fatty acid derivatives, aldehydes, other esters, and alcohols, reflecting higher amounts of all chemical classes during summer. This is consistent with previous reports showing increased plant volatile emissions in response to elevated temperature (warming). Examples include strong increasing effects of warming on terpenoids and other VOCs emission from Arctic ecosystems (Rinnan et al., 2020), stimulation of monoterpenes by night-time warming in heath ecosystems (Tiiva et al., 2017), and increased emissions of major volatile classes by the *L. scoparium* in association with high temperatures (Effah et al., 2020c). Other authors (e.g., Gouinguené and Turlings, 2002; Kivimäenpää et al., 2016; Kramshøj et al., 2016; Lindwall et al., 2016) have also reported positive effects of higher temperatures on VOC emissions.

Although emissions typically correlate positively with temperature, there may be a threshold at which emissions will decline. For instance, Gouinguené and Turlings (2002) found that total VOCs emissions of *Zea mays* decreased when temperature was increased from 27 to 37°C. Analogously, heat stress caused massive emissions from *Nicotiana tabacum* at temperatures 52–54°C, but a decline in emissions at 55°C (Turan et al., 2019). Temperature, therefore, can have varied effects on VOC emissions, as summarized below.

Elevated temperature stimulates both gross and net plant primary productivity, which coincides with augmented isoprene emissions as simulated by the photosynthetic-based isoprene scheme (Yue et al., 2015). This implies that temperature can regulate the availability of substrates required for VOCs synthesis. Photosynthetic products like pyruvate and glyceraldehyde-3-phosphate supply energy for the formation of isopentenyl diphosphate (IPP) and dimethylallyl diphosphate (DMAPP) from which isoprene and terpenes originate *via* the 2-C-methyl-D-erythritol 4-phosphate (MEP) and mevalonate (MVA) pathways (Dudareva et al., 2006). This makes photosynthetic products the main constraints for the production of volatile compounds *via* these pathways, and several studies have found a positive correlation between gross photosynthetic capacity and isoprene emission (Kuhn et al., 2004; Possell et al., 2004; Lahr et al., 2015).

High temperatures also directly affect biosynthetic enzymes and the expression of their associated VOCs regulatory genes, thus impacting emissions. For example, the isoprene synthase (IspS) activity responded strongly to temperature, with exponentially increasing activity between 15 and 40°C. IspS activity reached an optimum at 50°C and correlated strongly with VOC emission from *Quercus robur* (Lehning et al., 1999). Similarly, heat stress strongly induced IspS activity in *Populus alba* (Sasaki et al., 2005) and transgenic *Arabidopsis thaliana* plants (Sasaki et al., 2007), corresponding with higher emission levels. However, VOCs regulation by enzyme activities and related gene expressions is a complicated mechanism (Ibrahim et al., 2010) and could be species and compound dependant. For example, increasing night-time temperature led to variable expressions of the genes 1-deoxy-D-xylulose 5-phosphate reductoisomerase (DXR), 1-deoxy-D-xylulose 5-phosphate synthase (DXS), IPP, and hydroxymethylglutaryl CoA reductase in *Betula pendula* and *Populus tremula*, which coincided with the differences in volatile emissions (Ibrahim et al., 2010). Both day and night-time temperatures were significantly different between the sampling occasions in our study (Supplementary Figure 2) and could relate to the observed emissions.

Elevated temperature is linked to increased stomatal conductance (Urban et al., 2017) and can directly affect the diffusion rate of volatile compounds at a given time. Stomatal opening is expected to be accompanied by large bursts of emissions, mainly from storage pools (Niinemets et al., 2002; Hüve et al., 2007). However, the intensity of stomatal control on emissions is contingent on the pool size of volatiles stored in lipid and water phases (Harley, 2013), with noticeable control on more soluble compounds like alcohols and carboxylic acids compared to highly volatile compounds (Niinemets et al., 2002, 2004; Niinemets and Reichstein, 2003).

Temperature can also indirectly affect volatile emissions through its influence on different biotic and abiotic variables. For example, elevated temperature can amplify the severity of drought by enhancing soil moisture evaporation. In our study, higher temperatures in summer corresponded to relatively lower soil water content (Figure 5) and may account for the observed higher emissions in summer, as shown in earlier reports (Copolovici et al., 2014; Campbell et al., 2018). We indeed observed a strong negative relationship between soil moisture content and the emissions of aldehydes and other esters and a moderate negative relationship with sesquiterpenes (Figure 6). Another potential indirect effect of temperature on the variable emissions observed in this study could be the interaction between temperature and plant phenology. *D. subulatum*’s phenology would have been changing between the four sampling occasions. For example, flowering occurred from November to March. This covers the summer months in our study and may impact the composition of volatile blends measured during this period. Although we did not directly test this in the present study, higher temperatures have been shown to increase the floral volatiles from some plants, for instance (Hu et al., 2013; Farré-Armengol et al., 2014).

Other environmental factors like soil nutrient availability also affect plant volatile emissions. We found differences in K and P between seasons, with higher levels measured in ES (Figure 5), which coincide with the higher volatile emissions at this sampling time. We also found that increased K levels positively relate to the emissions of fatty acid derivatives, aldehydes, and other esters but negatively correlated with monoterpenoids and sesquiterpenes. On the other hand, high P levels were associated with increased emissions of monoterpenoids, other esters and sesquiterpenes (Figure 6). The mechanisms behind the relationships between soil nutrients and VOC emissions are poorly documented. Phosphorus, however, is crucial in terpenoid production because it is a significant component of ATP and NADPH required for terpenoid synthesis and an essential constituent of terpenoid precursors (IPP and DMAPP) (Ormeno and Fernandez, 2012). In comparison, nitrogen stimulates electron transport rate and photosynthesis, which supplies energy and carbon substrate for isoprenoid production (Ormeno and Fernandez, 2012). Like our results, other studies have reported a positive relationship between nutrient availability and VOC emissions. These include increased total emissions from *Z. mays* under high fertilization (Gouinguené and Turlings, 2002), enhancement of α-thujene, acetic acid and LOX compounds with increasing N availability from *Brassica napus* (Veromann et al., 2013) and induced higher emissions by infested conventional *B*. *napus* grown at a high-soil nutrient level (Ibrahim et al., 2008). Lower emission in nutrient-depleted soils is likely due to the lack of substrates required to synthesize compounds, as previously suggested by Gouinguené and Turlings (2002).

This study is a contribution to our understanding of VOC emissions of native plant species in the wild and the environmental factors impacting them, but other aspects require further research. Future studies should investigate the associations between plant phenology and its VOC emissions in nature. We also recommend more studies to explore the potential links between increased photosynthesis in warmer summers and VOC emissions. The potential effects of global warming on plant biochemical and physiological processes and their ecological impacts also deserve attention. Lastly, we acknowledge the possibility of our results being influenced by microbes and unidentified belowground processes and encourage future research to investigate the effect of such organisms and processes on VOC emissions over several seasons.

## Conclusion and Potential Ecological Impacts

For the first time, we characterized the VOCs emissions of New Zealand’s endemic *D. subulatum* and showed that this plant is a prolific emitter of terpenoids. Among the measured parameters, *D. subulatum*’s emissions showed a clear seasonal pattern with higher emissions during summer, mainly related to higher temperatures. Although other environmental variables such as soil moisture content and nutrients were associated with changes in emissions, the relationships were compound-dependent.

Climate change is expected to impact soil chemistry, air temperature, and plant development. Our results support the claim that the current warming trend is likely to increase plant volatile emissions globally and possibly affect their ecological roles (Peñuelas and Staudt, 2010; Rinnan et al., 2020). For instance, changes in emission in response to climate change can shape competitive outcomes for plants (Wang et al., 2010). Modification in emissions can also influence the communication between plants, herbivores and natural enemies of herbivores (Clavijo McCormick, 2016), which could be significant in shaping plant communities (Kegge and Pierik, 2010; Effah et al., 2019). Furthermore, volatile compounds including isoprene, monoterpenes, and green leaf volatiles play critical roles in improving thermotolerance, resistance to cold-stress, and serve as antioxidants (Holopainen, 2004; Zuo et al., 2017; Cofer et al., 2018). Therefore, further investigation is needed to establish whether altering emissions by climate change could enhance or impair VOCs’ protective functions.

Many volatile compounds also interfere with some atmospheric processes, reacting with O_3_, nitrogen oxides and hydroxyl radicals, and are linked to cloud formation (Blande et al., 2014; Zhao et al., 2017). The products of these reactions can be redeposited on plants and could affect plants communication with their environment and atmospheric quality. A recent study showed that α-pinene’s oxidation products are deposited on plants and re-emitted, affecting plants acceptability to herbivores (Mofikoya et al., 2020). This research area requires further investigation to increase our knowledge of the impact of climate-induced emissions on plant-plant, plant-insect, and plant-microbe interactions, especially in vulnerable and threatened native ecosystems.

## Data Availability Statement

The raw data supporting the conclusions of this article will be made available by the authors, without undue reservation.

## Author Contributions

EE and ACM conceived the project, collected the data, and led the writing of the manuscript. DB, PP, MP, and JH contributed to the final design and data collection. EE analyzed and interpreted the data with the advice of ACM. All authors were involved in editing and provided critical comments on the manuscript, and approved it for publication.

## Conflict of Interest

The authors declare that the research was conducted in the absence of any commercial or financial relationships that could be construed as a potential conflict of interest.

## Publisher’s Note

All claims expressed in this article are solely those of the authors and do not necessarily represent those of their affiliated organizations, or those of the publisher, the editors and the reviewers. Any product that may be evaluated in this article, or claim that may be made by its manufacturer, is not guaranteed or endorsed by the publisher.
