# Supplementary material for: Seasonal Volatile Emission Patterns of the Endemic New Zealand Shrub Dracophyllum subulatum on the North Island Central Plateau
Source: Front Plant Sci. 2021 Oct 15;12:734531. doi: 10.3389/fpls.2021.734531 (PMC8553956; doi:10.3389/fpls.2021.734531)
Supplement: Supplementary file 1 [file Data_Sheet_1.pdf]

Supplementary Material

Table S1. Location of experimental sites on the central plateau, New Zealand. The table also shows the number of plants sampled at each site (*n*).

Site	Dominant woody species	*n*	Location
1	*Dracophyllum*	10	Long. 175.73705 – Lat. -39.3112166
2	*Dracophyllum* and mānuka	5	Long. 175.685483 – Lat. - 39.432933
3	*Dracophyllum* and heather	5	Long. 175.68785 – Lat. -39.4206
4	*Dracophyllum* and broom	5	Long. 175.737466 – Lat. -39.315116

Table S2. Linear discriminant analysis (LDA) loadings for the volatile compounds emitted by *Dracophyllum* in early summer, late summer, late autumn, and winter.

Compound	LD1	LD2	LD3
(*E*)-2-hexenal	54.98329	36.36279	21.06329
(*E*)-2-hexenyl acetate	-4.23816	-54.2449	2.799437
(*E*)-α-bergamotene	13.52548	-3.93856	-6.52653
(*E*)-β-caryophyllene	-7.28718	0.127925	-0.78996
(*Z*)-2-hexenyl acetate	-11.8821	8.969216	4.541337
(*Z*)-3-hexenol	-13.4642	-9.05311	10.29689
(*Z*)-3-hexenyl acetate	-2.61092	9.868331	-2.63745
(*Z*)-β-ocimene	7.122515	0.869792	19.37962
3-methyl-1-butanol acetate	34.11499	-24.554	18.97154
Aromadendrene	-10.5113	12.90716	13.74117
Copaene	-13.1654	14.67991	1.283036
Decanal	53.4427	-35.6049	47.84412
Dodecanal	-9.1717	-32.9654	-61.8321
Eremophilene	-13.4916	12.41306	13.3705
Ethyl hexanoate	-173.928	5.792355	-21.3946
Ethyl octanoate	-162.611	-0.39093	113.4305
Germacrene D	1.984006	2.589042	4.524341
Heptanal	-75.8737	-9.91541	33.96285

Table S2 (continued)

**Compound**	**LD1**	**LD2**	**LD3**
Hexanol	36.88221	60.18157	-100.327
Hexyl 2-methylbutyrate	152.1955	-5.19865	46.61819
Hexyl acetate	35.16706	-13.2497	2.656033
Humulene	16.55101	-9.9527	-2.12746
Isoledene	2.561271	37.74345	-0.54822
Lemonol	-6.86166	-1.36948	10.12916
Limonene	22.30649	-29.558	-2.41444
Linalool	8.601385	-5.18884	4.656085
Linalyl acetate	-15.6054	9.445585	-0.68026
Nonanal	-4.06591	-3.20439	27.55938
Nonanol	-21.0578	36.69631	14.52538
Octanal	-10.3268	13.20678	-49.0417
Perillene	40.02628	-17.0246	-12.3876
Octanol	-45.4844	-38.8307	21.57211
Phenethyl acetate	-105.763	88.71951	-81.2211
Valencene	-13.3654	9.332224	8.661065
Sabinene	-41.4596	31.98924	-14.2971
Zingiberene	-3.61826	-27.7578	67.85428
α-amorphene	-10.3469	9.054637	4.675114
α-bourbonene	5.300976	-9.68983	-5.36959
α-cubebene	-10.635	-26.5905	-47.8779
(*E*,*E*)-α-farnesene	6.681426	5.658401	1.409786
α-panasinsene	26.06639	-38.5065	-26.7892
α-pinene	22.91256	-18.0853	10.65297
β-cubebene	5.968427	14.27398	-5.11256
β-pinene	9.181619	14.07895	7.3255
γ-cadinene	1.976978	13.96715	0.99481
δ-cadinene	5.413849	-23.6103	-13.3428

Table S3. Summary of generalized linear models indicating the effects of different parameters on the major chemical classes identified from *Dracophyllum*.

	n_eff	Rhat	mean	mcse	sd	2.50%	25%	50%	75%	97.50%
**Fatty acid derivatives**										
(Intercept)	15,040	1.00015	0.37567	0.0002	0.02448	0.32754	0.35929	0.37552	0.39199	0.42418
Temperature	14,914	0.99995	0.25789	0.00022	0.02734	0.20421	0.23962	0.25813	0.27617	0.31144
SWC	15,031	0.99996	-0.00952	0.00025	0.03048	-0.0698	-0.03006	-0.0097	0.01093	0.05041
Phosphorus	14,795	0.99992	-0.00892	0.00027	0.03295	-0.07368	-0.03086	-0.0088	0.01337	0.0555
Potassium	15,037	0.99997	0.05543	0.00028	0.03477	-0.01234	0.03239	0.05529	0.07879	0.1241
sigma	14,869	1.00002	0.24437	0.00015	0.0178	0.21209	0.23195	0.2434	0.25573	0.28207
mean_PPD	14,737	1.00001	0.37562	0.00029	0.03481	0.30644	0.35224	0.37562	0.39889	0.44465
log-posterior	15,006	0.99997	-7.13275	0.01457	1.78512	-11.4212	-8.07496	-6.78893	-5.82908	-4.68951
**Monoterpenoids**										
(Intercept)	14,857	0.99994	0.1096	0.00025	0.03027	0.05011	0.08949	0.10966	0.12993	0.16902
Temperature	14,453	0.99986	0.0982	0.00028	0.03365	0.03332	0.07542	0.09839	0.12049	0.16479
SWC	14,392	1.00006	0.0228	0.00031	0.03772	-0.05037	-0.00273	0.02255	0.04835	0.0983
Phosphorus	14,675	1.00001	0.09316	0.00034	0.04075	0.01242	0.06608	0.09349	0.12017	0.17347
Potassium	14,747	0.99998	-0.06967	0.00035	0.04283	-0.15428	-0.09854	-0.06976	-0.04072	0.01349
sigma	14,794	0.99986	0.30216	0.00018	0.02206	0.26282	0.28677	0.30082	0.31604	0.34922
mean_PPD	14,774	1.00004	0.10948	0.00035	0.04315	0.02463	0.08018	0.10934	0.13838	0.19423
log-posterior	14,985	0.99988	-28.3798	0.0146	1.78695	-32.7747	-29.33	-28.0197	-27.0641	-25.9468
**Sesquiterpenoids**										
(Intercept)	14,510	1.00037	0.33611	0.00025	0.02961	0.27811	0.31652	0.33573	0.35646	0.39387
Temperature	15,026	0.99988	0.20843	0.00027	0.03353	0.14214	0.18634	0.20856	0.23079	0.27401
SWC	15,304	1.00018	-0.03808	0.0003	0.03736	-0.11009	-0.0631	-0.03846	-0.01345	0.03621
Phosphorus	15,315	0.99987	0.03905	0.00033	0.04074	-0.04006	0.01143	0.03892	0.06631	0.1187
Potassium	14,923	1.00009	-0.02678	0.00035	0.04232	-0.10979	-0.05485	-0.02687	0.00115	0.05654
sigma	15,146	0.99985	0.2989	0.00018	0.02187	0.25954	0.28367	0.29761	0.31276	0.34495
mean_PPD	15,187	1.00006	0.33604	0.00034	0.04196	0.25389	0.30782	0.33615	0.36404	0.41877
log-posterior	14,500	1.00017	-27.3048	0.0146	1.75831	-31.4725	-28.2535	-26.9874	-26.0084	-24.8663
**Aldehydes**										
(Intercept)	15,210	0.99997	0.05952	0.00006	0.00686	0.0462	0.0549	0.05952	0.06417	0.07288
Temperature	14,491	0.99985	0.01447	0.00006	0.00764	-0.00044	0.00943	0.01443	0.01963	0.0296
SWC	15,187	1.00009	-0.01798	0.00007	0.00859	-0.035	-0.02367	-0.018	-0.01226	-0.00111
Phosphorus	14,896	1.00007	0.00603	0.00008	0.00929	-0.01234	-0.00012	0.00602	0.01224	0.02426
Potassium	14,555	1.0002	0.01422	0.00008	0.00979	-0.00501	0.00768	0.01423	0.02076	0.0336
sigma	14,664	1	0.06852	0.00004	0.00505	0.05955	0.06502	0.06819	0.07169	0.0794
mean_PPD	15,028	0.99997	0.05952	0.00008	0.00964	0.04045	0.053	0.0596	0.06602	0.0786
log-posterior	14,961	0.99992	119.925	0.01472	1.8009	115.5953	118.9755	120.2605	121.2519	122.3767

Table S3 (continued)

	n_eff	Rhat	mean	mcse	sd	2.50%	25%	50%	75%	97.50%
**Other esters**										
(Intercept)	14,754	1.00004	0.07429	0.00007	0.00904	0.05645	0.06831	0.07428	0.08029	0.09227
Temperature	15,093	0.99992	0.03987	0.00008	0.01018	0.01995	0.03303	0.03986	0.04682	0.0595
SWC	14,740	0.99996	-0.03827	0.00009	0.01138	-0.06063	-0.04576	-0.03828	-0.03066	-0.01598
Phosphorus	15,012	1.00022	0.03408	0.0001	0.01235	0.00976	0.02584	0.03409	0.04237	0.05821
Potassium	14,652	0.99992	0.03753	0.00011	0.01294	0.01226	0.02863	0.03746	0.04616	0.06336
sigma	14,765	0.99991	0.09059	0.00006	0.00672	0.07856	0.08589	0.09017	0.09492	0.10503
mean_PPD	15,274	0.9999	0.07434	0.0001	0.01279	0.04918	0.06586	0.07414	0.08296	0.09966
log-posterior	14,904	1.00012	92.13277	0.01473	1.79851	87.79341	91.173	92.47267	93.46228	94.58986
**Other alcohols**										
(Intercept)	14,437	0.99991	0.01124	0.00002	0.00242	0.00645	0.00963	0.01125	0.01288	0.01596
Temperature	14,888	1.00005	0.01112	0.00002	0.0027	0.00587	0.00929	0.01112	0.01292	0.01641
SWC	15,146	0.99994	0.00177	0.00002	0.00299	-0.00416	-0.00021	0.00179	0.00376	0.00751
Phosphorus	15,379	0.99996	-0.00329	0.00003	0.00324	-0.00952	-0.0055	-0.0033	-0.00114	0.00312
Potassium	15,400	0.99994	-0.00095	0.00003	0.00344	-0.00774	-0.00324	-0.00089	0.00134	0.00568
sigma	15,135	1.00003	0.02414	0.00001	0.00177	0.02097	0.02289	0.02403	0.02528	0.02793
mean_PPD	14,832	1.00006	0.01126	0.00003	0.00339	0.0046	0.00898	0.01126	0.01355	0.0179
log-posterior	14,778	1.00017	224.2428	0.01461	1.77597	219.9144	223.3053	224.5724	225.5446	226.6957

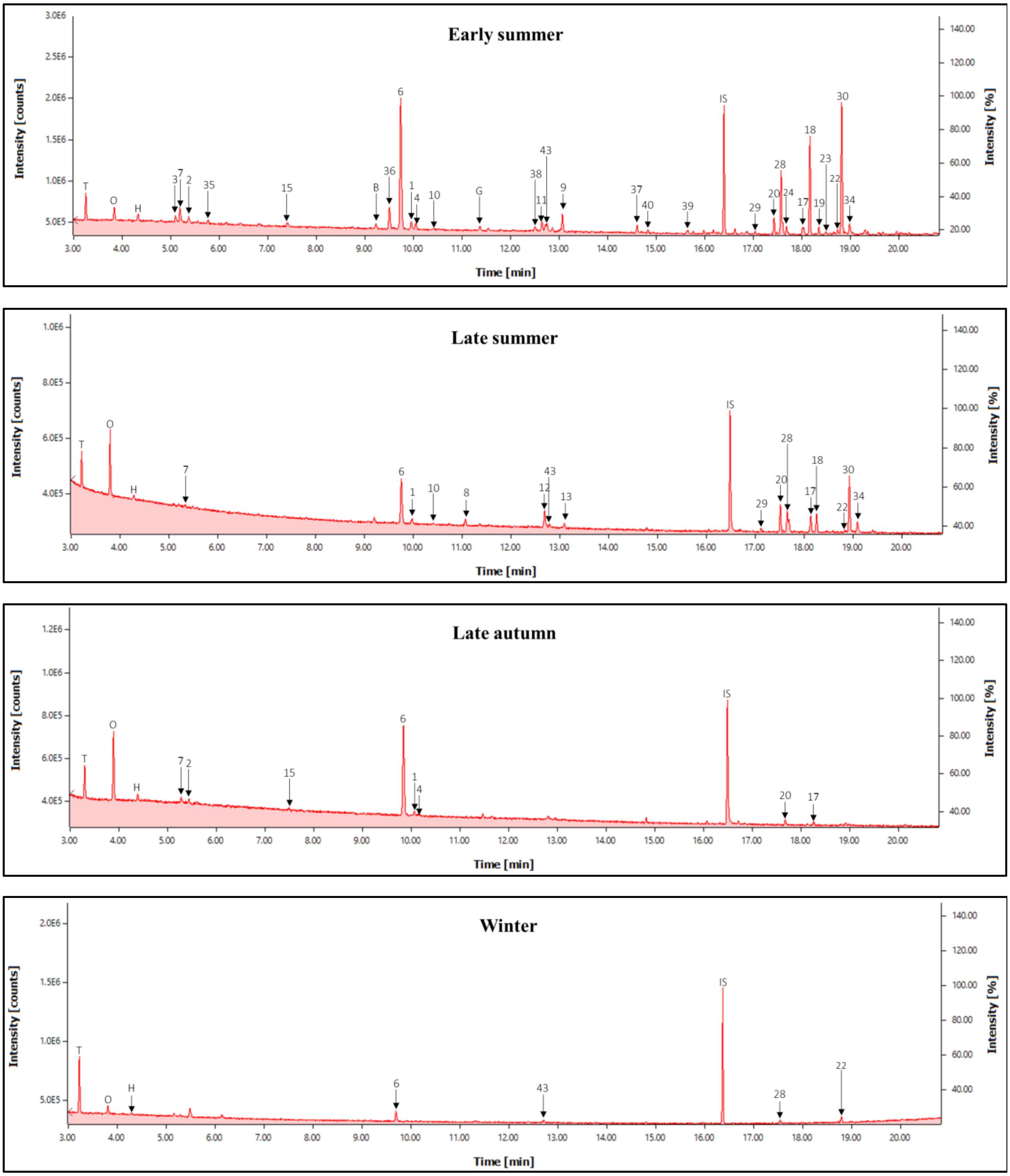

Figure S1. Chromatogram from the same *D. subulatum* plant on different sampling occasions. Numbers correspond to the compounds identified in Table 1 in the manuscript as follows; (1) hexyl acetate, (2) hexanol, (3) (*E*)-2-hexenal, (4) (*E*)-2-hexenyl acetate, (6) (*Z*)-3-hexenyl acetate, (7) (*Z*)-3-hexenol, (8) (*Z*)-β-ocimene, (9) lemonol, (10) limonene, (11) linalool, (12) linalyl acetate, (13) perillene, (15) α-pinene, (17) (*E*)-α-bergamotene, (18) (*E*)-β-caryophyllene, (19) aromadendrene, (20) copaene, (22) germacrene D, (23) humulene, (24) isoledene,

(28) α-bourbonene, (29) α-cubebene, (30) (*E*,*E*)-α-farnesene, (34) δ-cadinene, (35) 3-methyl-1-butanol acetate, (36) ethyl hexanoate, (37) ethyl octanoate, (38) hexyl 2-methylbutyrate, (39) phenethyl acetate, (40) decanal, (43) nonanal. Some unlabelled peaks are either other hydrocarbons that were also found in blank samples (e.g., Toluene (T), octane (O) and Hexane, 2,4-dimethyl (H)) or compounds identified in only a few plants (e.g., β-myrcene (B) and γ-terpinene (G)) and were excluded from the analysis.

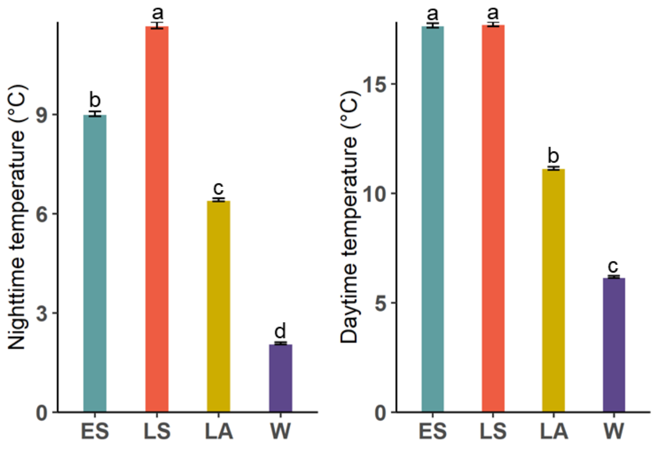

Figure S2. Average night-time and daytime temperatures for study sites in early summer (ES), late summer (LS), late autumn (LA) and winter (W). Night- and daytime data were extracted from loggers based on sunrise and sunset times for the months in respective sampling occasions. The data was analysed using the Kruskal-Wallis test, followed by the Mann Whitney test for pairwise comparisons. Different letters show significant differences between groups.
